# Some Parameterized Quantum Midpoint and Quantum Trapezoid Type Inequalities for Convex Functions with Applications

**DOI:** 10.3390/e23080996

**Published:** 2021-07-31

**Authors:** Suphawat Asawasamrit, Muhammad Aamir Ali, Sotiris K. Ntouyas, Jessada Tariboon

**Affiliations:** 1 Intelligent and Nonlinear Dynamic Innovations Research Center, Department of Mathematics, Faculty of Applied Science, King Mongkut’s University of Technology North Bangkok, Bangkok 10800, Thailand; suphawat.a@sci.kmutnb.ac.th; 2 Jiangsu Key Laboratory for NSLSCS, School of Mathematical Sciences, Nanjing Normal University, Nanjing 210023, China; 3 Department of Mathematics, University of Ioannina, 451 10 Ioannina, Greece; sntouyas@uoi.gr; 4 Nonlinear Analysis and Applied Mathematics (NAAM)-Research Group, Department of Mathematics, Faculty of Science, King Abdulaziz University, P.O. Box 80203, Jeddah 21589, Saudi Arabia

**Keywords:** Hermite–Hadamard inequality, midpoint and trapezoid inequalities, *q*-calculus, convex functions

## Abstract

Quantum information theory, an interdisciplinary field that includes computer science, information theory, philosophy, cryptography, and entropy, has various applications for quantum calculus. Inequalities and entropy functions have a strong association with convex functions. In this study, we prove quantum midpoint type inequalities, quantum trapezoidal type inequalities, and the quantum Simpson’s type inequality for differentiable convex functions using a new parameterized *q*-integral equality. The newly formed inequalities are also proven to be generalizations of previously existing inequities. Finally, using the newly established inequalities, we present some applications for quadrature formulas.

## 1. Introduction

In convex functions theory, Hermite–Hadamard (HH) inequality is very important and was discovered by C. Hermite and J. Hadamard independently (see also [1] and [2] (p. 137)),

(1) $$F\left(\frac{\;\pi_{1}+\;\pi_{2}}{2}\right)\leq \frac{1}{\;\pi_{2}-\;\pi_{1}}\int_{\;\pi_{1}}^{\;\pi_{2}}F\left(\;\nu \right)d\;\nu \leq \frac{F\left(\;\pi_{1}\right)+F\left(\;\pi_{2}\right)}{2}$$

where F is a convex function. In the case of concave mappings, the above inequality is satisfied in reverse order.

In [3], Kirmaci proved the following inequality connected to the left-side of inequality (1).

**Theorem** **1.** *For a mapping F:I⊂R→R, which is differentiable on $I^{\circ }$, $\;\pi_{1},\;\pi_{2}\in I^{\circ },$$\;\pi_{1}<\;\pi_{2}$ with $\left|F^{\prime }\right|$ convex on $\left[\;\pi_{1},\;\pi_{2}\right]$, then*

(2) $$\left|\frac{1}{\;\pi_{2}-\;\pi_{1}}\int_{\;\pi_{1}}^{\;\pi_{2}}F\left(\;\nu \right)d\;\nu -F\left(\frac{\;\pi_{1}+\;\pi_{2}}{2}\right)\right|\leq \frac{\left(\;\pi_{2}-\;\pi_{1}\right)}{8}\left[\left|F^{\prime }\left(\;\pi_{1}\right)\right|+\left|F^{\prime }\left(\;\pi_{2}\right)\right|\right].$$

In [4], the authors proved the following inequality linked to the right part of inequality (1).

**Theorem** **2.** *For a mapping F:I⊂R→R, which is differentiable on $I^{\circ }$, $\;\pi_{1},\;\pi_{2}\in I^{\circ },$$\;\pi_{1}<\;\pi_{2}$ with $\left|F^{\prime }\right|$ convex on $\left[\;\pi_{1},\;\pi_{2}\right]$, then*

(3) $$\left|\frac{F\left(\;\pi_{1}\right)+F\left(\;\pi_{2}\right)}{2}-\frac{1}{\;\pi_{2}-\;\pi_{1}}\int_{\;\pi_{1}}^{\;\pi_{2}}F\left(\;\nu \right)d\;\nu \right|\leq \frac{\left(\;\pi_{2}-\;\pi_{1}\right)}{8}\left[\left|F^{\prime }\left(\;\pi_{1}\right)\right|+\left|F^{\prime }\left(\;\pi_{2}\right)\right|\right].$$

Sarikaya et al. [5] proved the following Simpson’s type inequalities for differentiable convex functions.

**Theorem** **3.** *For a mapping F:I⊂R→R, which is differentiable on $I^{\circ }$, $\;\pi_{1},\;\pi_{2}\in I^{\circ },$$\;\pi_{1}<\;\pi_{2}$ with $\left|F^{\prime }\right|$ convex on $\left[\;\pi_{1},\;\pi_{2}\right]$, then*

(4) $$\begin{array}{ccc} & & \left|\frac{1}{6}\left[F\left(\;\pi_{1}\right)+4F\left(\frac{\;\pi_{1}+\;\pi_{2}}{2}\right)+F\left(\;\pi_{2}\right)\right]-\frac{1}{\;\pi_{2}-\;\pi_{1}}\int_{\;\pi_{1}}^{\;\pi_{2}}F\left(\;\nu \right)d\;\nu \right|\\ & \leq & \frac{5\left(\;\pi_{2}-\;\pi_{1}\right)}{72}\left[\left|F^{\prime }\left(\;\pi_{1}\right)\right|+\left|F^{\prime }\left(\;\pi_{2}\right)\right|\right]. \end{array}$$

**Theorem** **4.** *For a mapping F:I⊂R→R, which is differentiable on $I^{\circ }$, $\;\pi_{1},\;\pi_{2}\in I^{\circ }$ with $\;\pi_{1}<\;\pi_{2}$ and if ${\left|F^{\prime }\right|}^{p}$, p≥1 is a convex mapping on $\left[\;\pi_{1},\;\pi_{2}\right]$, then the following inequality holds:*

(5) $$\begin{array}{ccc} & & \left|\frac{1}{6}\left[F\left(\;\pi_{1}\right)+4F\left(\frac{\;\pi_{1}+\;\pi_{2}}{2}\right)+F\left(\;\pi_{2}\right)\right]-\frac{1}{\;\pi_{2}-\;\pi_{1}}\int_{\;\pi_{1}}^{\;\pi_{2}}F\left(\;\nu \right)d\;\nu \right|\\ & \leq & \frac{\left(\;\pi_{2}-\;\pi_{1}\right)}{72}{\left(5\right)}^{1-\frac{1}{p}}\left\{{\left[\frac{29{\left|F^{\prime }\left(\;\pi_{1}\right)\right|}^{p}+61{\left|F^{\prime }\left(\;\pi_{2}\right)\right|}^{p}}{18}\right]}^{\frac{1}{p}}+{\left[\frac{61{\left|F^{\prime }\left(\;\pi_{1}\right)\right|}^{p}+29{\left|F^{\prime }\left(\;\pi_{2}\right)\right|}^{p}}{18}\right]}^{\frac{1}{p}}\right\}. \end{array}$$

On the other hand, several studies have been carried out in the domain of *q*-analysis; beginning with Euler, the quantum computation of *q*-calculus, which is regarded as a relationship between physics and mathematics, must be studied in order to gain proficiency in mathematics. It has a wide range of applications in mathematics, including in combinatorics, simple hypergeometric functions, number theory, orthogonal polynomials, and other sciences, as well as mechanics, relativity theory, and quantum theory [6,7]. Quantum calculus also has many applications in quantum information theory, which is an interdisciplinary area that encompasses computer science, information theory, philosophy, and cryptography, among other areas [8,9,10]. Euler is thought to be the inventor of this significant branch of mathematics. He used the *q*-parameter in Newton’s work on infinite series. Later, Jackson presented *q*-calculus, which is also known as no-limits calculus, in a methodical manner [11,12]. In 1966, Al-Salam [13] introduced a *q*-analogue of the *q*-fractional integral and *q*-Riemann–Liouville fractional. Since then, the amount of related research has gradually increased. In particular, in 2013, Tariboon and Ntouyas introduced the ${}_{\;\pi_{1}}D_{q}$-difference operator and $q_{\;\pi_{1}}$-integral in [14]. In 2020, Bermudo et al. introduced the notion of the ${}^{\;\pi_{2}}D_{q}$ derivative and $q^{\;\pi_{2}}$-integral in [15]. Sadjang generalized this to quantum calculus and introduced the notions of post-quantum calculus or shortly $\left(p,q\right)$-calculus in [16]. In [17], Tunç and Göv gave the post-quantum variant of the ${}_{a}D_{q}$-difference operator and $q_{a}$-integral. Recently, in 2021, Chu et al. introduced the notions of the ${}^{b}D_{p,q}$ derivative and ${\left(p,q\right)}^{b}$-integral in [18].

Many integral inequalities have been studied using quantum integrals for various types of functions. For example, in [15,19,20,21,22,23,24,25], the authors used ${}_{\;\pi_{1}}D_{q},{\;}^{\;\pi_{2}}D_{q}$-derivatives and $q_{\;\pi_{1}},q^{\;\pi_{2}}$-integrals to prove HH integral inequalities and their left–right estimates for convex and coordinated convex functions. In [26], Noor et al. presented a generalized version of quantum HH integral inequalities. For generalized quasi-convex functions, Nwaeze et al. proved certain parameterized quantum integral inequalities in [27]. Khan et al. proved quantum HH inequality using the Green function in [28]. Budak et al. [29], Ali et al. [30,31], and Vivas-Cortez et al. [32] developed new quantum Simpson’s and quantum Newton’s type inequalities for convex and coordinated convex functions. For quantum Ostrowski’s inequalities for convex and co-ordinated convex functions, one can consult [33,34,35]. Kunt et al. [36] generalized the results of [21] and proved HH type inequalities and their left estimates using the ${}_{a}D_{p,q}$-difference operator and ${\left(p,q\right)}_{a}$-integral. Recently, Latif et al. [37] found the right estimates of HH type inequalities proved by Kunt et al. [36]. To prove Ostrowski’s inequalities, Chu et al. [18] used the concepts of the ${}^{b}D_{p,q}$-difference operator and ${\left(p,q\right)}^{b}$-integral. Recently, Vivas-Cortez et al. [38] generalized the results of [15] and proved HH type inequalities and their left estimates using the ${}^{b}D_{p,q}$-difference operator and ${\left(p,q\right)}^{b}$-integral.

Inspired by these ongoing studies, we establish a generalized form of quantum midpoint and quantum trapezoid type inequalities; these newly established inequalities are the generalizations of inequalities (2)–(5) and the inequalities proved in the work by Sarikaya et al. [39].

The structure of this paper is as follows: a brief overview of the concepts of *q*-calculus, as well as some related works, is given in Section 2. In Section 3, we show the relationship between the results presented here and comparable results in the literature by proving some new quantum integral inequalities. We present some applications of quadrature formulas in Section 4. Section 5 concludes with some recommendations for future studies.

## 2. Preliminaries of *q*-Calculus and Some Inequalities

In this section, we recall some basic concepts about *q*-calculus and integral inequalities in this area. Further, here and in the following, we use $q\in \left(0,1\right)$ and the following notation (see [7]):

$${\left[n\right]}_{q}=\frac{1-q^{n}}{1-q}=1+q+q^{2}+\ldots +q^{n-1},\;\;q\in \left(0,1\right).$$

In [12], from 0 to $\pi_{2}$, Jackson gave the *q*-Jackson integral as follows:

(6) $$\int_{0}^{\;\pi_{2}}F\left(\;\nu \right)\begin{array}{c} d_{q}\;\nu \end{array}=\left(1-q\right)\;\pi_{2}\sum_{n=0}^{\infty }q^{n}F\left(\;\pi_{2}q^{n}\right)$$

provided the sum converges absolutely.

**Definition** **1** ([14])**.** *The $q_{\;\pi_{1}}$-derivative of a mapping $F:\left[\;\pi_{1},\;\pi_{2}\right]\to R$ at $\;\nu \in \left[\;\pi_{1},\;\pi_{2}\right]$ is defined as*

(7) $$\begin{array}{c} {}_{\;\pi_{1}}D_{q}F\left(\;\nu \right) \end{array}=\frac{F\left(\;\nu \right)-F\left(q\;\nu +\left(1-q\right)\;\pi_{1}\right)}{\left(1-q\right)\left(\;\nu -\;\pi_{1}\right)},\;\;\nu \neq \;\pi_{1}.$$

*If $\;\nu =\;\pi_{1}$, we define ${}_{\;\pi_{1}}D_{q}F\left(\;\pi_{1}\right)=\lim_{\;\nu \to \;\pi_{1}}\begin{array}{c} {}_{\;\pi_{1}}D_{q}F\left(\;\nu \right) \end{array}$ if it exists and it is finite.*

**Definition** **2** ([15])**.** *The $q^{\;\pi_{2}}$-derivative of a mapping $F:\left[\;\pi_{1},\;\pi_{2}\right]\to R$ at $\;\nu \in \left[\;\pi_{1},\;\pi_{2}\right]$ is defined as*

$$\begin{array}{c} {}^{\;\pi_{2}}D_{q}F\left(\;\nu \right) \end{array}=\frac{F\left(q\;\nu +\left(1-q\right)\;\pi_{2}\right)-F\left(\;\nu \right)}{\left(1-q\right)\left(\;\pi_{2}-\;\nu \right)},\;\;\nu \neq \;\pi_{2}.$$

*If $\nu =\pi_{2}$, we define ${}^{\;\pi_{2}}D_{q}F\left(\pi_{2}\right)=\lim_{\;\nu \to \;\pi_{2}}\begin{array}{c} {}^{\;\pi_{2}}D_{q}F\left(\;\nu \right) \end{array}$ if it exists and it is finite.*

**Definition** **3** ([14])**.** *The $q_{\;\pi_{1}}$-integral of a mapping $F:\left[\;\pi_{1},\;\pi_{2}\right]\to R$ is defined as*

$$\int_{\;\pi_{1}}^{\;\nu }F\left(\;\mu \right)\begin{array}{c} {}_{\;\pi_{1}}d_{q}\;\mu \end{array}=\left(1-q\right)\left(\;\nu -\;\pi_{1}\right)\sum_{n=0}^{\infty }q^{n}F\left(q^{n}\;\nu +\left(1-q^{n}\right)\;\pi_{1}\right),$$

*where $\;\nu \in \left[\;\pi_{1},\;\pi_{2}\right].$*

**Definition** **4** ([15])**.** *The $q^{\;\pi_{2}}$-integral of a mapping $F:\left[\;\pi_{1},\;\pi_{2}\right]\to R$ is defined as*

$$\int_{\;\nu }^{\;\pi_{2}}F\left(\;\mu \right)\begin{array}{c} {}^{\;\pi_{2}}d_{q}\;\mu \end{array}=\left(1-q\right)\left(\;\pi_{2}-\;\nu \right)\sum_{n=0}^{\infty }q^{n}F\left(q^{n}\;\nu +\left(1-q^{n}\right)\;\pi_{2}\right),$$

*where $\;\nu \in \left[\;\pi_{1},\;\pi_{2}\right].$*

In the literature, we have the following two different quantum variants of the HH inequality (1) (see [15,21]).

**Theorem** **5.** *For the convex mapping $\;F:\left[\;\pi_{1},\;\pi_{2}\right]\to R$, the following inequalities are valid:*

(8) $$\;F\left(\frac{q\;\pi_{1}+\;\pi_{2}}{{\left[2\right]}_{q}}\right)\leq \frac{1}{\;\pi_{2}-\;\pi_{1}}\int_{\;\pi_{1}}^{\;\pi_{2}}\;F\left(\;\nu \right){\;}_{\;\pi_{1}}d_{q}\;\nu \leq \frac{q\;F\left(\;\pi_{1}\right)+\;F\left(\;\pi_{2}\right)}{{\left[2\right]}_{q}}$$

*and*

(9) $$\;F\left(\frac{\;\pi_{1}+q\;\pi_{2}}{{\left[2\right]}_{q}}\right)\leq \frac{1}{\;\pi_{2}-\;\pi_{1}}\int_{\;\pi_{1}}^{\;\pi_{2}}\;F\left(\;\nu \right){\;}^{\;\pi_{2}}d_{q}\;\nu \leq \frac{\;F\left(\;\pi_{1}\right)+q\;F\left(\;\pi_{2}\right)}{{\left[2\right]}_{q}}.$$

Recently, Budak [40] proved the following quantum variants of inequalities (2) and (3) linked to inequality (9).

**Theorem** **6.** *For a mapping F:I⊂R→R, q-differentiable on $I^{\circ }$, $\;\pi_{1},\;\pi_{2}\in I^{\circ },$$\;\pi_{1}<\;\pi_{2}$ with $\left|{}^{\;\pi_{2}}D_{q}\;F\right|$ convex mapping on $\left[\;\pi_{1},\;\pi_{2}\right]$, then*

$$\begin{array}{ccc} & & \left|\frac{\;F\left(\;\pi_{1}\right)+q\;F\left(\;\pi_{2}\right)}{{\left[2\right]}_{q}}-\frac{1}{\;\pi_{2}-\;\pi_{1}}\int_{\;\pi_{1}}^{\;\pi_{2}}\;F\left(\;\nu \right){\;}^{\;\pi_{2}}d_{q}\;\nu \right|\\ & \leq & \left(\;\pi_{2}-\;\pi_{1}\right)\left[\frac{q^{2}\left(1+4q+q^{2}\right)}{{\left[3\right]}_{q}{\left[2\right]}_{q}^{4}}\left|{}^{\;\pi_{2}}D_{q}\;F\left(\;\pi_{1}\right)\right|+\frac{q^{2}\left(1+3q^{2}+2q^{3}\right)}{{\left[3\right]}_{q}{\left[2\right]}_{q}^{4}}\left|{}^{\;\pi_{2}}D_{q}\;F\left(\;\pi_{2}\right)\right|\right] \end{array}$$

*and*

(10) $$\begin{array}{ccc} & & \left|\frac{1}{\;\pi_{2}-\;\pi_{1}}\int_{\;\pi_{1}}^{\;\pi_{2}}\;F\left(\;\nu \right){\;}^{\;\pi_{2}}d_{q}\;\nu -\;F\left(\frac{\;\pi_{1}+q\;\pi_{2}}{{\left[2\right]}_{q}}\right)\right|\\ & \leq & q\left(\;\pi_{2}-\;\pi_{1}\right)\left[\frac{3}{{\left[3\right]}_{q}{\left[2\right]}_{q}^{3}}\left|{}^{\;\pi_{2}}D_{q}\;F\left(\;\pi_{1}\right)\right|+\frac{2q{\left[2\right]}_{q}-1}{{\left[3\right]}_{q}{\left[2\right]}_{q}^{3}}\left|{}^{\;\pi_{2}}D_{q}\;F\left(\;\pi_{2}\right)\right|\right]. \end{array}$$

## 3. Main Results

In this section, we prove, for *q*-differentiable convex functions, some *q*-integral inequalities of midpoint and trapezoidal formula types.

**Lemma** **1.** *Let $F:\left[\;\pi_{1},\;\pi_{2}\right]\to R$ be a $q^{\;\pi_{2}}$-differentiable functions such that ${}^{\;\pi_{2}}D_{q}F$ is integrable and $\;\varsigma \in \left[0,1\right]$. Then, we have*

(11) $$\begin{array}{ccc} & & {\left[2\right]}_{q}\;\varsigma \frac{F\left(\;\pi_{1}\right)+qF\left(\;\pi_{2}\right)}{{\left[2\right]}_{q}}-\left({\left[2\right]}_{q}\;\varsigma -1\right)F\left(\frac{\;\pi_{1}+q\;\pi_{2}}{{\left[2\right]}_{q}}\right)-\frac{1}{\;\pi_{2}-\;\pi_{1}}\int_{\;\pi_{1}}^{\;\pi_{2}}F\left(\;\nu \right){\;}^{\;\pi_{2}}d_{q}\;\nu \\ & = & \left(\;\pi_{2}-\;\pi_{1}\right)\left[\int_{0}^{\frac{1}{{\left[2\right]}_{q}}}q\left(\;\varsigma -\;\mu \right){\;}^{\;\pi_{2}}D_{q}F\left(\;\mu \;\pi_{1}+\left(1-\;\mu \right)\;\pi_{2}\right)d_{q}\;\mu \right.\\ & & \left.+\int_{\frac{1}{{\left[2\right]}_{q}}}^{1}\left(1-\;\varsigma -q\;\mu \right){\;}^{\;\pi_{2}}D_{q}F\left(\;\mu \;\pi_{1}+\left(1-\;\mu \right)\;\pi_{2}\right)d_{q}\;\mu \right]. \end{array}$$

**Proof.**  From the fundamental concepts of *q*-integrals, we have

(12) $$\begin{array}{ccc} & & \left(\;\pi_{2}-\;\pi_{1}\right)\left[\int_{0}^{\frac{1}{{\left[2\right]}_{q}}}q\left(\;\varsigma -\;\mu \right){\;}^{\;\pi_{2}}D_{q}F\left(\;\mu \;\pi_{1}+\left(1-\;\mu \right)\;\pi_{2}\right)d_{q}\;\mu \right.\\ & & \left.+\int_{\frac{1}{{\left[2\right]}_{q}}}^{1}\left(1-\;\varsigma -q\;\mu \right){\;}^{\;\pi_{2}}D_{q}F\left(\;\mu \;\pi_{1}+\left(1-\;\mu \right)\;\pi_{2}\right)d_{q}\;\mu \right]\\ & = & \left(\;\pi_{2}-\;\pi_{1}\right)\left[\int_{0}^{\frac{1}{{\left[2\right]}_{q}}}\left({\left[2\right]}_{q}\;\varsigma -1\right){\;}^{\;\pi_{2}}D_{q}F\left(\;\mu \;\pi_{1}+\left(1-\;\mu \right)\;\pi_{2}\right)d_{q}\;\mu \right.\\ & & \left.+\int_{0}^{1}\left(1-\;\varsigma -q\;\mu \right){\;}^{\;\pi_{2}}D_{q}F\left(\;\mu \;\pi_{1}+\left(1-\;\mu \right)\;\pi_{2}\right)d_{q}\;\mu \right]\\ & = & \left(\;\pi_{2}-\;\pi_{1}\right)\left[I_{1}+I_{2}\right]. \end{array}$$

Definitions 2 and 4 give us the following:

$$\begin{array}{ccc} I_{1} & = & \left({\left[2\right]}_{q}\;\varsigma -1\right)\int_{0}^{\frac{1}{{\left[2\right]}_{q}}}{\;}^{\;\pi_{2}}D_{q}F\left(\;\mu \;\pi_{1}+\left(1-\;\mu \right)\;\pi_{2}\right)d_{q}\;\mu \\ & = & \left({\left[2\right]}_{q}\;\varsigma -1\right)\int_{0}^{\frac{1}{{\left[2\right]}_{q}}}\frac{F\left(q\;\mu \;\pi_{1}+\left(1-q\;\mu \right)\;\pi_{2}\right)-F\left(\;\mu \;\pi_{1}+\left(1-\;\mu \right)\;\pi_{2}\right)}{\left(1-q\right)\;\mu \left(\;\pi_{2}-\;\pi_{1}\right)}d_{q}\;\mu \\ & = & \frac{\left({\left[2\right]}_{q}\;\varsigma -1\right)}{\;\pi_{2}-\;\pi_{1}}\left[\sum_{n=0}^{\infty }F\left(\frac{q^{n+1}}{{\left[2\right]}_{q}}\;\pi_{1}+\left(1-\frac{q^{n+1}}{{\left[2\right]}_{q}}\right)\;\pi_{2}\right)-\sum_{n=0}^{\infty }F\left(\frac{q^{n}}{{\left[2\right]}_{q}}\;\pi_{1}+\left(1-\frac{q^{n}}{{\left[2\right]}_{q}}\right)\;\pi_{2}\right)\right]\\ & = & \frac{\left({\left[2\right]}_{q}\;\varsigma -1\right)}{\;\pi_{2}-\;\pi_{1}}\left[F\left(\;\pi_{2}\right)-F\left(\frac{\;\pi_{1}+q\;\pi_{2}}{{\left[2\right]}_{q}}\right)\right] \end{array}$$

and

$$\begin{array}{ccc} I_{2} & = & \left(1-\;\varsigma \right)\int_{0}^{1}{\;}^{\;\pi_{2}}D_{q}F\left(\;\mu \;\pi_{1}+\left(1-\;\mu \right)\;\pi_{2}\right)d_{q}\;\mu -q\int_{0}^{1}\;\mu {\;}^{\;\pi_{2}}D_{q}F\left(\;\mu \;\pi_{1}+\left(1-\;\mu \right)\;\pi_{2}\right)d_{q}\;\mu \\ & = & \frac{\left(1-\;\varsigma \right)}{\;\pi_{2}-\;\pi_{1}}\left[F\left(\;\pi_{2}\right)-F\left(\;\pi_{1}\right)\right]-\left[\frac{1}{{\left(\;\pi_{2}-\;\pi_{1}\right)}^{2}}\int_{\;\pi_{1}}^{\;\pi_{2}}F\left(\;\nu \right){\;}^{\;\pi_{2}}d_{q}\;\nu -\frac{1}{\;\pi_{2}-\;\pi_{1}}F\left(\;\pi_{1}\right)\right]. \end{array}$$

By putting the computed values of $I_{1}$ and $I_{2}$ in equality (12), we obtain the resultant equality (11). □

**Remark** **1.** *If we take the limit as $q\to 1^{-}$ in Lemma 1, then it becomes* [39], *Lemma 1.*

**Theorem** **7.** *Under the conditions of Lemma 1, if $\left|{\;}^{\;\pi_{2}}D_{q}F\right|$ is a convex mapping, then*

(13) $$\begin{array}{ccc} & & \left|{\left[2\right]}_{q}\;\varsigma \frac{F\left(\;\pi_{1}\right)+qF\left(\;\pi_{2}\right)}{{\left[2\right]}_{q}}-\left({\left[2\right]}_{q}\;\varsigma -1\right)F\left(\frac{\;\pi_{1}+q\;\pi_{2}}{{\left[2\right]}_{q}}\right)-\frac{1}{\;\pi_{2}-\;\pi_{1}}\int_{\;\pi_{1}}^{\;\pi_{2}}F\left(\;\nu \right){\;}^{\;\pi_{2}}d_{q}\;\nu \right|\\ & \leq & \left(\;\pi_{2}-\;\pi_{1}\right)\left\{\begin{array}{c} A_{1}\left(q;\;\varsigma \right)\left|{\;}^{\;\pi_{2}}D_{q}F\left(\;\pi_{1}\right)\right|+B_{1}\left(q;\;\varsigma \right)\left|{\;}^{\;\pi_{2}}D_{q}F\left(\;\pi_{2}\right)\right|,\;\;if\;0\leq \;\varsigma <\frac{1}{{\left[2\right]}_{q}},\\ A_{2}\left(q;\;\varsigma \right)\left|{\;}^{\;\pi_{2}}D_{q}F\left(\;\pi_{1}\right)\right|+B_{2}\left(q;\;\varsigma \right)\left|{\;}^{\;\pi_{2}}D_{q}F\left(\;\pi_{2}\right)\right|,\;\;if\;\frac{1}{{\left[2\right]}_{q}}\leq \;\varsigma \leq 1, \end{array}\right. \end{array}$$

*where*

$$\begin{array}{ccc} A_{1}\left(q;\;\varsigma \right) & = & \left[\frac{q}{{\left[2\right]}_{q}^{3}{\left[3\right]}_{q}}-\frac{q\;\varsigma }{{\left[2\right]}_{q}^{3}}+2\;\varsigma^{3}\frac{q^{3}}{{\left[2\right]}_{q}{\left[3\right]}_{q}}+2\frac{{\left(1-\;\varsigma \right)}^{3}}{{\left[2\right]}_{q}{\left[3\right]}_{q}}\right.\\ & & \left.+\left(\;\varsigma -1\right)\left(\frac{1}{{\left[2\right]}_{q}^{3}}+\frac{1}{{\left[2\right]}_{q}}\right)+\frac{q}{{\left[3\right]}_{q}}\left(1+\frac{1}{{\left[2\right]}_{q}^{3}}\right)\right],\\ B_{1}\left(q;\;\varsigma \right) & = & \left[\frac{q}{{\left[2\right]}_{q}^{3}}-\frac{q}{{\left[2\right]}_{q}^{3}{\left[3\right]}_{q}}+2\;\varsigma^{2}\frac{q^{2}}{{\left[2\right]}_{q}}-2\;\varsigma^{3}\frac{q^{3}}{{\left[2\right]}_{q}{\left[3\right]}_{q}}\right.\\ & & +2\frac{{\left(1-\;\varsigma \right)}^{2}}{{\left[2\right]}_{q}}+\frac{1}{{\left[2\right]}_{q}}\left(\;\varsigma +q-1\right)+\left(\;\varsigma -1\right)+\frac{q}{{\left[2\right]}_{q}^{3}}-2\frac{{\left(1-\;\varsigma \right)}^{3}}{{\left[2\right]}_{q}{\left[3\right]}_{q}}\\ & & \left.-\left(\left(\;\varsigma -1\right)\left(\frac{1}{{\left[2\right]}_{q}^{3}}+\frac{1}{{\left[2\right]}_{q}}\right)+\frac{q}{{\left[3\right]}_{q}}\left(1+\frac{1}{{\left[2\right]}_{q}^{3}}\right)\right)\right], \end{array}$$

$$A_{2}\left(q;\;\varsigma \right)=\frac{q}{{\left[3\right]}_{q}}-\frac{2q}{{\left[2\right]}_{q}^{3}{\left[3\right]}_{q}}-\frac{1}{{\left[2\right]}_{q}}+\frac{1}{{\left[2\right]}_{q}^{3}}+\frac{\;\varsigma }{{\left[2\right]}_{q}}$$

*and*

$$B_{2}\left(q;\;\varsigma \right)=\frac{1}{{\left[2\right]}_{q}}\left(q+2-\;\varsigma \right)+\frac{2q}{{\left[2\right]}_{q}^{3}{\left[3\right]}_{q}}+\left(\;\varsigma -1\right)-\frac{2q+1}{{\left[2\right]}_{q}^{3}}-\frac{q}{{\left[3\right]}_{q}}.$$

**Proof.**  Using the properties of the modulus, after taking the modulus in Lemma 1, we can obtain

(14) $$\begin{array}{ccc} & & \left|{\left[2\right]}_{q}\;\varsigma \frac{F\left(\;\pi_{1}\right)+qF\left(\;\pi_{2}\right)}{{\left[2\right]}_{q}}-\left({\left[2\right]}_{q}\;\varsigma -1\right)F\left(\frac{\;\pi_{1}+q\;\pi_{2}}{{\left[2\right]}_{q}}\right)-\frac{1}{\;\pi_{2}-\;\pi_{1}}\int_{\;\pi_{1}}^{\;\pi_{2}}F\left(\;\nu \right){\;}^{\;\pi_{2}}d_{q}\;\nu \right|\\ & \leq & \left(\;\pi_{2}-\;\pi_{1}\right)\left[q\int_{0}^{\frac{1}{{\left[2\right]}_{q}}}\left|\;\varsigma -\;\mu \right|\;\left|{}^{\;\pi_{2}}D_{q}F\left(\;\mu \;\pi_{1}+\left(1-\;\mu \right)\;\pi_{2}\right)\right|d_{q}\;\mu \right.\\ & & \left.+\int_{\frac{1}{{\left[2\right]}_{q}}}^{1}\left|1-\;\varsigma -q\;\mu \right|\;\left|{}^{\;\pi_{2}}D_{q}F\left(\;\mu \;\pi_{1}+\left(1-\;\mu \right)\;\pi_{2}\right)\right|d_{q}\;\mu \right]. \end{array}$$

Since $\left|{\;}^{\;\pi_{2}}D_{q}F\right|$ is a convex mapping, we therefore have

$$\begin{array}{ccc} & & \left|{\left[2\right]}_{q}\;\varsigma \frac{F\left(\;\pi_{1}\right)+qF\left(\;\pi_{2}\right)}{{\left[2\right]}_{q}}-\left({\left[2\right]}_{q}\;\varsigma -1\right)F\left(\frac{\;\pi_{1}+q\;\pi_{2}}{{\left[2\right]}_{q}}\right)-\frac{1}{\;\pi_{2}-\;\pi_{1}}\int_{\;\pi_{1}}^{\;\pi_{2}}F\left(\;\nu \right){\;}^{\;\pi_{2}}d_{q}\;\nu \right|\\ & \leq & \left(\;\pi_{2}-\;\pi_{1}\right)\left[q\int_{0}^{\frac{1}{{\left[2\right]}_{q}}}\left|\;\varsigma -\;\mu \right|\left[\;\mu \left|{\;}^{\;\pi_{2}}D_{q}F\left(\;\pi_{1}\right)\right|+\left(1-\;\mu \right)\left|{\;}^{\;\pi_{2}}D_{q}F\left(\;\pi_{2}\right)\right|\right]d_{q}\;\mu \right.\\ & & \left.+\int_{\frac{1}{{\left[2\right]}_{q}}}^{1}\left|1-\;\varsigma -q\;\mu \right|\left[\;\mu \left|{\;}^{\;\pi_{2}}D_{q}F\left(\;\pi_{1}\right)\right|+\left(1-\;\mu \right)\left|{\;}^{\;\pi_{2}}D_{q}F\left(\;\pi_{2}\right)\right|\right]d_{q}\;\mu \right]\\ & = & \left|{\;}^{\;\pi_{2}}D_{q}F\left(\;\pi_{1}\right)\right|\left[q\int_{0}^{\frac{1}{{\left[2\right]}_{q}}}\;\mu \left|\;\varsigma -\;\mu \right|d_{q}\;\mu +\int_{\frac{1}{{\left[2\right]}_{q}}}^{1}\;\mu \left|1-\;\varsigma -q\;\mu \right|d_{q}\;\mu \right]\\ & & +\left|{\;}^{\;\pi_{2}}D_{q}F\left(\;\pi_{2}\right)\right|\left[q\int_{0}^{\frac{1}{{\left[2\right]}_{q}}}\left(1-\;\mu \right)\left|\;\varsigma -\;\mu \right|d_{q}\;\mu +\int_{\frac{1}{{\left[2\right]}_{q}}}^{1}\left(1-\;\mu \right)\left|1-\;\varsigma -q\;\mu \right|d_{q}\;\mu \right]. \end{array}$$

One can easily observe that for $0\leq \;\varsigma <\frac{1}{2},$

$$\begin{array}{ccc} & & \int_{0}^{\frac{1}{{\left[2\right]}_{q}}}q\;\mu \left|\;\varsigma -\;\mu \right|d_{q}\;\mu +\int_{\frac{1}{{\left[2\right]}_{q}}}^{1}\;\mu \left|1-\;\varsigma -q\;\mu \right|d_{q}\;\mu \\ & = & \int_{0}^{\;\varsigma }q\;\mu \left(\;\varsigma -\;\mu \right)d_{q}\;\mu +\int_{\;\varsigma }^{\frac{1}{{\left[2\right]}_{q}}}q\;\mu \left(\;\mu -\;\varsigma \right)d_{q}\;\mu \\ & & +\int_{\frac{1}{{\left[2\right]}_{q}}}^{\frac{1-\;\varsigma }{q}}\;\mu \left(1-\;\varsigma -q\;\mu \right)d_{q}\;\mu +\int_{\frac{1-\;\varsigma }{q}}^{1}\;\mu \left(-1+\;\varsigma +q\;\mu \right)d_{q}\;\mu \\ & = & A_{1}\left(q;\;\varsigma \right),\\ & & \int_{0}^{\frac{1}{{\left[2\right]}_{q}}\;}q\left(1-\;\mu \right)\left|\;\varsigma -\;\mu \right|d_{q}\;\mu +\int_{\frac{1}{{\left[2\right]}_{q}}}^{1}\left(1-\;\mu \right)\left|1-\;\varsigma -q\;\mu \right|d_{q}\;\mu \\ & = & \int_{0}^{\;\varsigma }q\left(1-\;\mu \right)\left(\;\varsigma -\;\mu \right)d_{q}\;\mu +\int_{\;\varsigma }^{\frac{1}{{\left[2\right]}_{q}}}q\left(1-\;\mu \right)\left(\;\mu -\;\varsigma \right)d_{q}\;\mu \\ & & +\int_{\frac{1}{{\left[2\right]}_{q}}}^{\frac{1-\;\varsigma }{q}}\left(1-\;\mu \right)\left(1-\;\varsigma -q\;\mu \right)d_{q}\;\mu +\int_{\frac{1-\;\varsigma }{q}}^{1}\left(1-\;\mu \right)\left(-1+\;\varsigma +q\;\mu \right)d_{q}\;\mu \\ & = & B_{1}\left(q;\;\varsigma \right). \end{array}$$

Now, for $\frac{1}{{\left[2\right]}_{q}}\leq \;\varsigma \leq 1$, one can see

$$\begin{array}{ccc} & & \int_{0}^{\frac{1}{{\left[2\right]}_{q}}}q\;\mu \left|\;\varsigma -\;\mu \right|d_{q}\;\mu +\int_{\frac{1}{{\left[2\right]}_{q}}}^{1}\;\mu \left|1-\;\varsigma -q\;\mu \right|d_{q}\;\mu \\ & = & \int_{0}^{\frac{1}{{\left[2\right]}_{q}}}q\;\mu \left(\;\varsigma -\;\mu \right)d_{q}\;\mu +\int_{\frac{1}{{\left[2\right]}_{q}}}^{1}\;\mu \left(-1+\;\varsigma +q\;\mu \right)d_{q}\;\mu \\ & = & A_{2}\left(q;\;\varsigma \right) \end{array}$$

and

$$\begin{array}{ccc} & & \int_{0}^{\frac{1}{{\left[2\right]}_{q}}}q\left(1-\;\mu \right)\left|\;\varsigma -\;\mu \right|d_{q}\;\mu +\int_{\frac{1}{{\left[2\right]}_{q}}}^{1}\left(1-\;\mu \right)\left|1-\;\varsigma -q\;\mu \right|d_{q}\;\mu \\ & = & \int_{0}^{\frac{1}{{\left[2\right]}_{q}}}q\left(1-\;\mu \right)\left(\;\varsigma -\;\mu \right)d_{q}\;\mu +\int_{\frac{1}{{\left[2\right]}_{q}}}^{1}\left(1-\;\mu \right)\left(-1+\;\varsigma +q\;\mu \right)d_{q}\;\mu \\ & = & B_{2}\left(q;\;\varsigma \right) \end{array}$$

Thus, the proof is finished. □

**Remark** **2.** *Setting the limit as $q\to 1^{-}$ in Theorem 7, then it reduces to [39], Theorem 5.*

**Corollary** **1.** *The inequality (13) in Theorem 7 reduces to the following quantum trapezoid type inequality by assuming $\;\varsigma =\frac{1}{{\left[2\right]}_{q}}$:*

$$\begin{array}{ccc} & & \left|\frac{F\left(\;\pi_{1}\right)+qF\left(\;\pi_{2}\right)}{{\left[2\right]}_{q}}-\frac{1}{\;\pi_{2}-\;\pi_{1}}\int_{\;\pi_{1}}^{\;\pi_{2}}F\left(\;\nu \right){\;}^{\;\pi_{2}}d_{q}\;\nu \right|\\ & \leq & \left(\;\pi_{2}-\;\pi_{1}\right)\left[A_{2}\left(q;\frac{1}{{\left[2\right]}_{q}}\right)\left|{\;}^{\;\pi_{2}}D_{q}F\left(\;\pi_{1}\right)\right|+B_{2}\left(q;\frac{1}{{\left[2\right]}_{q}}\right)\left|{\;}^{\;\pi_{2}}D_{q}F\left(\;\pi_{2}\right)\right|\right]. \end{array}$$

**Remark** **3.** *The inequality (13) in Theorem 7 reduces to (10) by assuming ς=0.*

**Remark** **4.** *In Corollary 1, if we set the limit as $q\to 1^{-}$, then we obtain the inequality (2).*

**Remark** **5.** *In Theorem 7, if we set the limit as $q\to 1^{-}$ and later assume ς=0, then we obtain the inequality (3).*

**Corollary** **2.** *In Theorem 7, if we set $\;\varsigma =\frac{1}{{\left[6\right]}_{q}}$, then we obtain the following quantum Simpson’s inequality:*

$$\begin{array}{ccc} & & \left|\frac{1}{{\left[6\right]}_{q}}\left[F\left(\;\pi_{1}\right)+\frac{q^{2}}{{\left[4\right]}_{q}}F\left(\frac{\;\pi_{1}+q\;\pi_{2}}{{\left[2\right]}_{q}}\right)+qF\left(\;\pi_{2}\right)\right]-\int_{\;\pi_{1}}^{\;\pi_{2}}F\left(\;\nu \right){\;}^{\;\pi_{2}}d_{q}\;\nu \right|\\ & \leq & \left(\;\pi_{2}-\;\pi_{1}\right)\left[A_{1}\left(q;\frac{1}{{\left[6\right]}_{q}}\right)\left|{\;}^{\;\pi_{2}}D_{q}F\left(\;\pi_{1}\right)\right|+B_{1}\left(q;\frac{1}{{\left[6\right]}_{q}}\right)\left|{\;}^{\;\pi_{2}}D_{q}F\left(\;\pi_{2}\right)\right|\right]. \end{array}$$

**Remark** **6.** *If we take the limit as $q\to 1^{-}$ in Corollary 2, then we recapture the inequality (4).*

**Theorem** **8.** *Under the conditions of Lemma 1, if ${\left|{\;}^{\;\pi_{2}}D_{q}F\right|}^{p},$ as p≥1 is a convex mapping, then*

(*i*) *If $0\leq \;\varsigma <\frac{1}{{\left[2\right]}_{q}},$ then*
  $$\begin{array}{ccc} & & \left|{\left[2\right]}_{q}\;\varsigma \frac{F\left(\;\pi_{1}\right)+qF\left(\;\pi_{2}\right)}{{\left[2\right]}_{q}}-\left({\left[2\right]}_{q}\;\varsigma -1\right)F\left(\frac{\;\pi_{1}+q\;\pi_{2}}{{\left[2\right]}_{q}}\right)-\frac{1}{\;\pi_{2}-\;\pi_{1}}\int_{\;\pi_{1}}^{\;\pi_{2}}F\left(\;\nu \right){\;}^{\;\pi_{2}}d_{q}\;\nu \right|\\ & \leq & \left(\;\pi_{2}-\;\pi_{1}\right)\left\{A_{3}^{1-\frac{1}{p}}\left(q;\;\varsigma \right){\left(B_{3}\left(q;\;\varsigma \right){\left|{\;}^{\;\pi_{2}}D_{q}F\left(\;\pi_{1}\right)\right|}^{p}+C_{1}\left(q;\;\varsigma \right){\left|{\;}^{\;\pi_{2}}D_{q}F\left(\;\pi_{2}\right)\right|}^{p}\right)}^{\frac{1}{p}}\right.\\ & & \left.+A_{4}^{1-\frac{1}{p}}\left(q;\;\varsigma \right){\left(B_{4}\left(q;\;\varsigma \right){\left|{\;}^{\;\pi_{2}}D_{q}F\left(\;\pi_{1}\right)\right|}^{p}+C_{2}\left(q;\;\varsigma \right){\left|{\;}^{\;\pi_{2}}D_{q}F\left(\;\pi_{2}\right)\right|}^{p}\right)}^{\frac{1}{p}}\right\}. \end{array}$$
(*ii*) *If $\frac{1}{{\left[2\right]}_{q}}\leq \;\varsigma \leq 1$, then*
  $$\begin{array}{ccc} & & \left|{\left[2\right]}_{q}\;\varsigma \frac{F\left(\;\pi_{1}\right)+qF\left(\;\pi_{2}\right)}{{\left[2\right]}_{q}}-\left({\left[2\right]}_{q}\;\varsigma -1\right)F\left(\frac{\;\pi_{1}+q\;\pi_{2}}{{\left[2\right]}_{q}}\right)-\frac{1}{\;\pi_{2}-\;\pi_{1}}\int_{\;\pi_{1}}^{\;\pi_{2}}F\left(\;\nu \right){\;}^{\;\pi_{2}}d_{q}\;\nu \right|\\ & \leq & \left(\;\pi_{2}-\;\pi_{1}\right)\left\{A_{5}^{1-\frac{1}{p}}\left(q;\;\varsigma \right){\left(B_{5}\left(q;\;\varsigma \right){\left|{\;}^{\;\pi_{2}}D_{q}F\left(\;\pi_{1}\right)\right|}^{p}+C_{3}\left(q;\;\varsigma \right){\left|{\;}^{\;\pi_{2}}D_{q}F\left(\;\pi_{2}\right)\right|}^{p}\right)}^{\frac{1}{p}}\right.\\ & & \left.+A_{6}^{1-\frac{1}{p}}\left(q;\;\varsigma \right){\left(B_{6}\left(q;\;\varsigma \right){\left|{\;}^{\;\pi_{2}}D_{q}F\left(\;\pi_{1}\right)\right|}^{p}+C_{4}\left(q;\;\varsigma \right){\left|{\;}^{\;\pi_{2}}D_{q}F\left(\;\pi_{2}\right)\right|}^{p}\right)}^{\frac{1}{p}}\right\}, \end{array}$$

*where*

$$\begin{array}{ccc} A_{3}\left(q;\;\varsigma \right) & = & \;\varsigma^{2}\frac{2q^{2}}{{\left[2\right]}_{q}}-\frac{\;\varsigma q}{{\left[2\right]}_{q}}+\frac{q}{{\left[2\right]}_{q}^{3}},\\ B_{3}\left(q;\;\varsigma \right) & = & \frac{q}{{\left[2\right]}_{q}^{3}{\left[3\right]}_{q}}-\frac{q\;\varsigma }{{\left[2\right]}_{q}^{3}}+2\;\varsigma^{3}\frac{q^{3}}{{\left[2\right]}_{q}{\left[3\right]}_{q}},\\ C_{1}\left(q;\;\varsigma \right) & = & \frac{q}{{\left[2\right]}_{q}^{3}}-\frac{q}{{\left[2\right]}_{q}^{3}{\left[3\right]}_{q}}+2\;\varsigma^{2}\frac{q^{2}}{{\left[2\right]}_{q}}-2\;\varsigma^{3}\frac{q^{3}}{{\left[2\right]}_{q}{\left[3\right]}_{q}},\\ A_{4}\left(q;\;\varsigma \right) & = & \frac{2{\left(1-\;\varsigma \right)}^{2}}{{\left[2\right]}_{q}}+\frac{1}{{\left[2\right]}_{q}}\left(q-1+\;\varsigma \right)+\frac{q}{{\left[2\right]}_{q}^{3}}+\;\varsigma -1,\\ B_{4}\left(q;\;\varsigma \right) & = & 2\frac{{\left(1-\;\varsigma \right)}^{3}}{{\left[2\right]}_{q}{\left[3\right]}_{q}}+\left(\;\varsigma -1\right)\left(\frac{1}{{\left[2\right]}_{q}^{3}}+\frac{1}{{\left[2\right]}_{q}}\right)+\frac{q}{{\left[3\right]}_{q}}\left(1+\frac{1}{{\left[2\right]}_{q}^{3}}\right)\\ C_{2}\left(q;\;\varsigma \right) & = & 2\frac{{\left(1-\;\varsigma \right)}^{2}}{{\left[2\right]}_{q}}+\frac{1}{{\left[2\right]}_{q}}\left(\;\varsigma +q-1\right)+\left(\;\varsigma -1\right)+\frac{q}{{\left[2\right]}_{q}^{3}}-2\frac{{\left(1-\;\varsigma \right)}^{3}}{{\left[2\right]}_{q}{\left[3\right]}_{q}}\\ & & -\left(\left(\;\varsigma -1\right)\left(\frac{1}{{\left[2\right]}_{q}^{3}}+\frac{1}{{\left[2\right]}_{q}}\right)+\frac{q}{{\left[3\right]}_{q}}\left(1+\frac{1}{{\left[2\right]}_{q}^{3}}\right)\right) \end{array}$$

*and*

$$\begin{array}{ccc} A_{5}\left(q;\;\varsigma \right) & = & \;\varsigma \frac{q}{{\left[2\right]}_{q}}-\frac{q}{{\left[2\right]}_{q}^{3}},\\ B_{5}\left(q;\;\varsigma \right) & = & \;\varsigma \frac{q}{{\left[2\right]}_{q}^{3}}-\frac{q}{{\left[2\right]}_{q}^{3}{\left[3\right]}_{q}},\\ C_{3}\left(q;\;\varsigma \right) & = & \;\varsigma \frac{q}{{\left[2\right]}_{q}}-\frac{q}{{\left[2\right]}_{q}^{3}}-\left(\;\varsigma \frac{q}{{\left[2\right]}_{q}^{3}}-\frac{q}{{\left[2\right]}_{q}^{3}{\left[3\right]}_{q}}\right),\\ A_{6}\left(q;\;\varsigma \right) & = & \frac{1}{{\left[2\right]}_{q}}\left(1+q-\;\varsigma \right)-\frac{q}{{\left[2\right]}_{q}^{3}}+\;\varsigma -1,\\ B_{6}\left(q;\;\varsigma \right) & = & \frac{q}{{\left[3\right]}_{q}}+\frac{1}{{\left[2\right]}_{q}}\left(\;\varsigma -1\right)+\frac{1-\;\varsigma {\left[2\right]}_{q}+\left(1-\;\varsigma \right)q^{2}}{{\left[2\right]}_{q}^{3}{\left[3\right]}_{q}}, \end{array}$$

$$\begin{array}{ccc} C_{4}\left(q;\;\varsigma \right) & = & \frac{1}{{\left[2\right]}_{q}}\left(1+q-\;\varsigma \right)-\frac{q}{{\left[2\right]}_{q}^{3}}+\;\varsigma -1\\ & & -\left(\frac{q}{{\left[3\right]}_{q}}+\frac{1}{{\left[2\right]}_{q}}\left(\;\varsigma -1\right)+\frac{1-\;\varsigma {\left[2\right]}_{q}+\left(1-\;\varsigma \right)q^{2}}{{\left[2\right]}_{q}^{3}{\left[3\right]}_{q}}\right). \end{array}$$

**Proof.**  Applying this to (14), the power mean inequality, we obtain

$$\begin{array}{ccc} & & \left|{\left[2\right]}_{q}\;\varsigma \frac{F\left(\;\pi_{1}\right)+qF\left(\;\pi_{2}\right)}{{\left[2\right]}_{q}}-\left({\left[2\right]}_{q}\;\varsigma -1\right)F\left(\frac{\;\pi_{1}+q\;\pi_{2}}{{\left[2\right]}_{q}}\right)-\frac{1}{\;\pi_{2}-\;\pi_{1}}\int_{\;\pi_{1}}^{\;\pi_{2}}F\left(\;\nu \right){\;}^{\;\pi_{2}}d_{q}\;\nu \right|\\ & \leq & \left(\;\pi_{2}-\;\pi_{1}\right)\left[{\left(\int_{0}^{\frac{1}{{\left[2\right]}_{q}}}q\left|\;\varsigma -\;\mu \right|d_{q}\;\mu \right)}^{1-\frac{1}{p}}{\left(\int_{0}^{\frac{1}{{\left[2\right]}_{q}}}q\left|\;\varsigma -\;\mu \right|\;{\left|{}^{\;\pi_{2}}D_{q}F\left(\;\mu \;\pi_{1}+\left(1-\;\mu \right)\;\pi_{2}\right)\right|}^{p}d_{q}\;\mu \right)}^{\frac{1}{p}}\right.\\ & & +{\left(\int_{\frac{1}{{\left[2\right]}_{q}}}^{1}\left|1-\;\varsigma -q\;\mu \right|d_{q}\;\mu \right)}^{1-\frac{1}{p}}{\left(\int_{\frac{1}{{\left[2\right]}_{q}}}^{1}\left|1-\;\varsigma -q\;\mu \right|\;{\left|{}^{\;\pi_{2}}D_{q}F\left(\;\mu \;\pi_{1}+\left(1-\;\mu \right)\;\pi_{2}\right)\right|}^{p}d_{q}\;\mu \right)}^{\frac{1}{p}}. \end{array}$$

Since ${\left|{\;}^{\;\pi_{2}}D_{q}F\right|}^{p},$ for p≥1, is convex, therefore, for $0\leq \;\varsigma \leq \frac{1}{{\left[2\right]}_{q}}$, we have

$$\begin{array}{ccc} & & {\left(\int_{0}^{\frac{1}{{\left[2\right]}_{q}}}q\left|\;\varsigma -\;\mu \right|d_{q}\;\mu \right)}^{1-\frac{1}{p}}{\left(\int_{0}^{\frac{1}{{\left[2\right]}_{q}}}q\left|\;\varsigma -\;\mu \right|\;{\left|{}^{\;\pi_{2}}D_{q}F\left(\;\mu \;\pi_{1}+\left(1-\;\mu \right)\;\pi_{2}\right)\right|}^{p}d_{q}\;\mu \right)}^{\frac{1}{p}}\\ & \leq & {\left(\int_{0}^{\frac{1}{{\left[2\right]}_{q}}}q\left|\;\varsigma -\;\mu \right|d_{q}\;\mu \right)}^{1-\frac{1}{p}}\\ & & \times {\left({\left|{\;}^{\;\pi_{2}}D_{q}F\left(\;\pi_{1}\right)\right|}^{p}\int_{0}^{\frac{1}{{\left[2\right]}_{q}}}q\;\mu \left|\;\varsigma -\;\mu \right|d_{q}\;\mu +{\left|{\;}^{\;\pi_{2}}D_{q}F\left(\;\pi_{2}\right)\right|}^{p}\int_{0}^{\frac{1}{{\left[2\right]}_{q}}}q\left(1-\;\mu \right)\left|\;\varsigma -\;\mu \right|d_{q}\;\mu \right)}^{\frac{1}{p}}\\ & = & {\left(\;\varsigma^{2}\frac{2q^{2}}{{\left[2\right]}_{q}}-\frac{\;\varsigma q}{{\left[2\right]}_{q}}+\frac{q}{{\left[2\right]}_{q}^{3}}\right)}^{1-\frac{1}{p}}\left({\left|{\;}^{\;\pi_{2}}D_{q}F\left(\;\pi_{1}\right)\right|}^{p}\left[\frac{q}{{\left[2\right]}_{q}^{3}{\left[3\right]}_{q}}-\frac{q\;\varsigma }{{\left[2\right]}_{q}^{3}}+2\;\varsigma^{3}\frac{q^{3}}{{\left[2\right]}_{q}{\left[3\right]}_{q}}\right]\right.\\ & & {\left.+{\left|{\;}^{\;\pi_{2}}D_{q}F\left(\;\pi_{2}\right)\right|}^{p}\left[\frac{q}{{\left[2\right]}_{q}^{3}}-\frac{q}{{\left[2\right]}_{q}^{3}{\left[3\right]}_{q}}+2\;\varsigma^{2}\frac{q^{2}}{{\left[2\right]}_{q}}-2\;\varsigma^{3}\frac{q^{3}}{{\left[2\right]}_{q}{\left[3\right]}_{q}}\right]\right)}^{\frac{1}{p}} \end{array}$$

and

$$\begin{array}{ccc} & & {\left(\int_{\frac{1}{{\left[2\right]}_{q}}}^{1}\left|1-\;\varsigma -q\;\mu \right|d_{q}\;\mu \right)}^{1-\frac{1}{p}}{\left(\int_{\frac{1}{{\left[2\right]}_{q}}}^{1}\left|1-\;\varsigma -q\;\mu \right|\;{\left|{}^{\;\pi_{2}}D_{q}F\left(\;\mu \;\pi_{1}+\left(1-\;\mu \right)\;\pi_{2}\right)\right|}^{p}d_{q}\;\mu \right)}^{\frac{1}{p}}\\ & \leq & {\left(\frac{2{\left(1-\;\varsigma \right)}^{2}}{{\left[2\right]}_{q}}+\frac{1}{{\left[2\right]}_{q}}\left(q-1+\;\varsigma \right)+\frac{q}{{\left[2\right]}_{q}^{3}}+\;\varsigma -1\right)}^{1-\frac{1}{p}}\\ & & \times \left({\left|{\;}^{\;\pi_{2}}D_{q}F\left(\;\pi_{1}\right)\right|}^{p}\left[2\frac{{\left(1-\;\varsigma \right)}^{3}}{{\left[2\right]}_{q}{\left[3\right]}_{q}}+\left(\;\varsigma -1\right)\left(\frac{1}{{\left[2\right]}_{q}^{3}}+\frac{1}{{\left[2\right]}_{q}}\right)+\frac{q}{{\left[3\right]}_{q}}\left(1+\frac{1}{{\left[2\right]}_{q}^{3}}\right)\right]\right.\\ & & +{\left|{\;}^{\;\pi_{2}}D_{q}F\left(\;\pi_{2}\right)\right|}^{p}\left[2\frac{{\left(1-\;\varsigma \right)}^{2}}{{\left[2\right]}_{q}}+\frac{1}{{\left[2\right]}_{q}}\left(\;\varsigma +q-1\right)+\left(\;\varsigma -1\right)+\frac{q}{{\left[2\right]}_{q}^{3}}-2\frac{{\left(1-\;\varsigma \right)}^{3}}{{\left[2\right]}_{q}{\left[3\right]}_{q}}\right.\\ & & {\left.\left.-\left(\left(\;\varsigma -1\right)\left(\frac{1}{{\left[2\right]}_{q}^{3}}+\frac{1}{{\left[2\right]}_{q}}\right)+\frac{q}{{\left[3\right]}_{q}}\left(1+\frac{1}{{\left[2\right]}_{q}^{3}}\right)\right)\right]\right)}^{\frac{1}{p}}. \end{array}$$

Similarly, for $\frac{1}{{\left[2\right]}_{q}}\leq \;\varsigma \leq 1$, we have

$$\begin{array}{ccc} & & {\left(\int_{0}^{\frac{1}{{\left[2\right]}_{q}}}q\left|\;\varsigma -\;\mu \right|d_{q}\;\mu \right)}^{1-\frac{1}{p}}{\left(\int_{0}^{\frac{1}{{\left[2\right]}_{q}}}q\left|\;\varsigma -\;\mu \right|\;{\left|{}^{\;\pi_{2}}D_{q}F\left(\;\mu \;\pi_{1}+\left(1-\;\mu \right)\;\pi_{2}\right)\right|}^{p}d_{q}\;\mu \right)}^{\frac{1}{p}}\\ & \leq & {\left(\int_{0}^{\frac{1}{{\left[2\right]}_{q}}}q\left|\;\varsigma -\;\mu \right|d_{q}\;\mu \right)}^{1-\frac{1}{p}}\\ & & \times {\left({\left|{\;}^{\;\pi_{2}}D_{q}F\left(\;\pi_{1}\right)\right|}^{p}\int_{0}^{\frac{1}{{\left[2\right]}_{q}}}q\;\mu \left|\;\varsigma -\;\mu \right|d_{q}\;\mu +{\left|{\;}^{\;\pi_{2}}D_{q}F\left(\;\pi_{2}\right)\right|}^{p}\int_{0}^{\frac{1}{{\left[2\right]}_{q}}}q\left(1-\;\mu \right)\left|\;\varsigma -\;\mu \right|d_{q}\;\mu \right)}^{\frac{1}{p}}\\ & = & {\left(\;\varsigma \frac{q}{{\left[2\right]}_{q}}-\frac{q}{{\left[2\right]}_{q}^{3}}\right)}^{1-\frac{1}{p}}\left({\left|{\;}^{\;\pi_{2}}D_{q}F\left(\;\pi_{1}\right)\right|}^{p}\left[\;\varsigma \frac{q}{{\left[2\right]}_{q}^{3}}-\frac{q}{{\left[2\right]}_{q}^{3}{\left[3\right]}_{q}}\right]\right.\\ & & {\left.+{\left|{\;}^{\;\pi_{2}}D_{q}F\left(\;\pi_{2}\right)\right|}^{p}\left[\;\varsigma \frac{q}{{\left[2\right]}_{q}}-\frac{q}{{\left[2\right]}_{q}^{3}}-\left(\;\varsigma \frac{q}{{\left[2\right]}_{q}^{3}}-\frac{q}{{\left[2\right]}_{q}^{3}{\left[3\right]}_{q}}\right)\right]\right)}^{\frac{1}{p}} \end{array}$$

and

$$\begin{array}{ccc} & & {\left(\int_{\frac{1}{{\left[2\right]}_{q}}}^{1}\left|1-\;\varsigma -q\;\mu \right|d_{q}\;\mu \right)}^{1-\frac{1}{p}}{\left(\int_{\frac{1}{{\left[2\right]}_{q}}}^{1}\left|1-\;\varsigma -q\;\mu \right|\;{\left|{}^{\;\pi_{2}}D_{q}F\left(\;\mu \;\pi_{1}+\left(1-\;\mu \right)\;\pi_{2}\right)\right|}^{p}d_{q}\;\mu \right)}^{\frac{1}{p}}\\ & \leq & {\left(\frac{1}{{\left[2\right]}_{q}}\left(1+q-\;\varsigma \right)-\frac{q}{{\left[2\right]}_{q}^{3}}+\;\varsigma -1\right)}^{1-\frac{1}{p}}\\ & & \times \left({\left|{\;}^{\;\pi_{2}}D_{q}F\left(\;\pi_{1}\right)\right|}^{p}\left[\frac{q}{{\left[3\right]}_{q}}+\frac{1}{{\left[2\right]}_{q}}\left(\;\varsigma -1\right)+\frac{1-\;\varsigma {\left[2\right]}_{q}+\left(1-\;\varsigma \right)q^{2}}{{\left[2\right]}_{q}^{3}{\left[3\right]}_{q}}\right]\right.\\ & & +{\left|{\;}^{\;\pi_{2}}D_{q}F\left(\;\pi_{2}\right)\right|}^{p}\left[\frac{1}{{\left[2\right]}_{q}}\left(1+q-\;\varsigma \right)-\frac{q}{{\left[2\right]}_{q}^{3}}+\;\varsigma -1\right.\\ & & {\left.\left.-\left(\frac{q}{{\left[3\right]}_{q}}+\frac{1}{{\left[2\right]}_{q}}\left(\;\varsigma -1\right)+\frac{1-\;\varsigma {\left[2\right]}_{q}+\left(1-\;\varsigma \right)q^{2}}{{\left[2\right]}_{q}^{3}{\left[3\right]}_{q}}\right)\right]\right)}^{\frac{1}{p}}. \end{array}$$

Therefore, the proof is finished. □

**Remark** **7.** *If we set the limit as $q\to 1^{-}$ in Theorem 8, then it reduces to [39], Theorem 6.*

**Corollary** **3.** *Theorem 8 reduces to the following new quantum trapezoid type inequality by assuming $\;\varsigma =\frac{1}{{\left[2\right]}_{q}}$:*

$$\begin{array}{ccc} & & \left|\frac{F\left(\;\pi_{1}\right)+qF\left(\;\pi_{2}\right)}{{\left[2\right]}_{q}}-\frac{1}{\;\pi_{2}-\;\pi_{1}}\int_{\;\pi_{1}}^{\;\pi_{2}}F\left(\;\nu \right){\;}^{\;\pi_{2}}d_{q}\;\nu \right|\\ & \leq & \left(\;\pi_{2}-\;\pi_{1}\right)\left\{A_{5}^{1-\frac{1}{p}}\left(q;\frac{1}{{\left[2\right]}_{q}}\right){\left(B_{5}\left(q;\frac{1}{{\left[2\right]}_{q}}\right){\left|{\;}^{\;\pi_{2}}D_{q}F\left(\;\pi_{1}\right)\right|}^{p}+C_{3}\left(q;\frac{1}{{\left[2\right]}_{q}}\right){\left|{\;}^{\;\pi_{2}}D_{q}F\left(\;\pi_{2}\right)\right|}^{p}\right)}^{\frac{1}{p}}\right.\\ & & \left.+A_{6}^{1-\frac{1}{p}}\left(q;\frac{1}{{\left[2\right]}_{q}}\right){\left(B_{6}\left(q;\frac{1}{{\left[2\right]}_{q}}\right){\left|{\;}^{\;\pi_{2}}D_{q}F\left(\;\pi_{1}\right)\right|}^{p}+C_{4}\left(q;\frac{1}{{\left[2\right]}_{q}}\right){\left|{\;}^{\;\pi_{2}}D_{q}F\left(\;\pi_{2}\right)\right|}^{p}\right)}^{\frac{1}{p}}\right\}. \end{array}$$

**Remark** **8.** *Theorem 8 reduces to [40], Theorem 2 (page 212) by assuming ς=0.*

**Corollary** **4.** *In Theorem 7, if we set $\;\varsigma =\frac{1}{{\left[6\right]}_{q}}$, then we obtain the following quantum Simpson’s inequality:*

$$\begin{array}{ccc} & & \left|\frac{1}{{\left[6\right]}_{q}}\left[F\left(\;\pi_{1}\right)+\frac{q^{2}}{{\left[4\right]}_{q}}F\left(\frac{\;\pi_{1}+q\;\pi_{2}}{{\left[2\right]}_{q}}\right)+qF\left(\;\pi_{2}\right)\right]-\int_{\;\pi_{1}}^{\;\pi_{2}}F\left(\;\nu \right){\;}^{\;\pi_{2}}d_{q}\;\nu \right|\\ & \leq & \left(\;\pi_{2}-\;\pi_{1}\right)\left\{A_{5}^{1-\frac{1}{p}}\left(q;\frac{1}{{\left[6\right]}_{q}}\right){\left(B_{5}\left(q;\frac{1}{{\left[6\right]}_{q}}\right){\left|{\;}^{\;\pi_{2}}D_{q}F\left(\;\pi_{1}\right)\right|}^{p}+C_{3}\left(q;\frac{1}{{\left[6\right]}_{q}}\right){\left|{\;}^{\;\pi_{2}}D_{q}F\left(\;\pi_{2}\right)\right|}^{p}\right)}^{\frac{1}{p}}\right.\\ & & \left.+A_{6}^{1-\frac{1}{p}}\left(q;\frac{1}{{\left[6\right]}_{q}}\right){\left(B_{6}\left(q;\frac{1}{{\left[6\right]}_{q}}\right){\left|{\;}^{\;\pi_{2}}D_{q}F\left(\;\pi_{1}\right)\right|}^{p}+C_{4}\left(q;\frac{1}{{\left[6\right]}_{q}}\right){\left|{\;}^{\;\pi_{2}}D_{q}F\left(\;\pi_{2}\right)\right|}^{p}\right)}^{\frac{1}{p}}\right\}. \end{array}$$

**Remark** **9.** *If we take the limit as $q\to 1^{-}$ in Corollary 4, then we recapture the inequality (5).*

## 4. Applications to Quadrature Rule

In this section, we present some applications of quadrature formulas using the results given in the last section.

**Proposition** **1.** *Under the assumptions of Theorem 7 with ς=1, we have:*

$$\begin{array}{ccc} & & \left|{\left[2\right]}_{q}\frac{F\left(\;\pi_{1}\right)+qF\left(\;\pi_{2}\right)}{{\left[2\right]}_{q}}-qF\left(\frac{\;\pi_{1}+q\;\pi_{2}}{{\left[2\right]}_{q}}\right)-\frac{1}{\;\pi_{2}-\;\pi_{1}}\int_{\;\pi_{1}}^{\;\pi_{2}}F\left(\;\nu \right){\;}^{\;\pi_{2}}d_{q}\;\nu \right|\\ & \leq & \left(\;\pi_{2}-\;\pi_{1}\right)\left[A_{2}\left(q;1\right)\left|{\;}^{\;\pi_{2}}D_{q}F\left(\;\pi_{1}\right)\right|+B_{2}\left(q;1\right)\left|{\;}^{\;\pi_{2}}D_{q}F\left(\;\pi_{2}\right)\right|\right]. \end{array}$$

**Proposition** **2.** *Under the assumptions of Theorem 7 with $\;\varsigma =\frac{1}{{\left[3\right]}_{q}}$, we have*

$$\begin{array}{ccc} & & \left|\frac{1}{{\left[3\right]}_{q}}\left[F\left(\;\pi_{1}\right)+q^{2}F\left(\frac{\;\pi_{1}+q\;\pi_{2}}{{\left[2\right]}_{q}}\right)+qF\left(\;\pi_{2}\right)\right]-\frac{1}{\;\pi_{2}-\;\pi_{1}}\int_{\;\pi_{1}}^{\;\pi_{2}}F\left(\;\nu \right){\;}^{\;\pi_{2}}d_{q}\;\nu \right|\\ & \leq & \left(\;\pi_{2}-\;\pi_{1}\right)\left[A_{1}\left(q;\frac{1}{{\left[3\right]}_{q}}\right)\left|{\;}^{\;\pi_{2}}D_{q}F\left(\;\pi_{1}\right)\right|+B_{1}\left(q;\frac{1}{{\left[3\right]}_{q}}\right)\left|{\;}^{\;\pi_{2}}D_{q}F\left(\;\pi_{2}\right)\right|\right]. \end{array}$$

**Proposition** **3.** *Under the assumptions of Theorem 7 with $\;\varsigma =\frac{1}{{\left[4\right]}_{q}}$, we have*

$$\begin{array}{ccc} & & \left|\frac{{\left[2\right]}_{q}}{{\left[4\right]}_{q}}\left[\frac{F\left(\;\pi_{1}\right)+qF\left(\;\pi_{2}\right)}{{\left[2\right]}_{q}}+q^{2}F\left(\frac{\;\pi_{1}+q\;\pi_{2}}{{\left[2\right]}_{q}}\right)\right]-\frac{1}{\;\pi_{2}-\;\pi_{1}}\int_{\;\pi_{1}}^{\;\pi_{2}}F\left(\;\nu \right){\;}^{\;\pi_{2}}d_{q}\;\nu \right|\\ & \leq & \left(\;\pi_{2}-\;\pi_{1}\right)\left[A_{1}\left(q;\frac{1}{{\left[4\right]}_{q}}\right)\left|{\;}^{\;\pi_{2}}D_{q}F\left(\;\pi_{1}\right)\right|+B_{1}\left(q;\frac{1}{{\left[4\right]}_{q}}\right)\left|{\;}^{\;\pi_{2}}D_{q}F\left(\;\pi_{2}\right)\right|\right]. \end{array}$$

**Proposition** **4.** *Under the assumptions of Theorem 8 with ς=1, we have*

$$\begin{array}{ccc} & & \left|{\left[2\right]}_{q}\frac{F\left(\;\pi_{1}\right)+qF\left(\;\pi_{2}\right)}{{\left[2\right]}_{q}}-qF\left(\frac{\;\pi_{1}+q\;\pi_{2}}{{\left[2\right]}_{q}}\right)-\frac{1}{\;\pi_{2}-\;\pi_{1}}\int_{\;\pi_{1}}^{\;\pi_{2}}F\left(\;\nu \right){\;}^{\;\pi_{2}}d_{q}\;\nu \right|\\ & \leq & \left(\;\pi_{2}-\;\pi_{1}\right)\left\{A_{5}^{1-\frac{1}{p}}\left(q;1\right){\left(B_{5}\left(q;1\right){\left|{\;}^{\;\pi_{2}}D_{q}F\left(\;\pi_{1}\right)\right|}^{p}+C_{3}\left(q;1\right){\left|{\;}^{\;\pi_{2}}D_{q}F\left(\;\pi_{2}\right)\right|}^{p}\right)}^{\frac{1}{p}}\right.\\ & & \left.+A_{6}^{1-\frac{1}{p}}\left(q;1\right){\left(B_{6}\left(q;1\right){\left|{\;}^{\;\pi_{2}}D_{q}F\left(\;\pi_{1}\right)\right|}^{p}+C_{4}\left(q;1\right){\left|{\;}^{\;\pi_{2}}D_{q}F\left(\;\pi_{2}\right)\right|}^{p}\right)}^{\frac{1}{p}}\right\}. \end{array}$$

**Proposition** **5.** *Under the assumptions of Theorem 8 with $\;\varsigma =\frac{1}{{\left[3\right]}_{q}}$, we have*

$$\begin{array}{ccc} & & \left|\frac{1}{{\left[3\right]}_{q}}\left[F\left(\;\pi_{1}\right)+q^{2}F\left(\frac{\;\pi_{1}+q\;\pi_{2}}{{\left[2\right]}_{q}}\right)+qF\left(\;\pi_{2}\right)\right]-\frac{1}{\;\pi_{2}-\;\pi_{1}}\int_{\;\pi_{1}}^{\;\pi_{2}}F\left(\;\nu \right){\;}^{\;\pi_{2}}d_{q}\;\nu \right|\\ & \leq & \left(\;\pi_{2}-\;\pi_{1}\right)\left\{A_{3}^{1-\frac{1}{p}}\left(q;\frac{1}{{\left[3\right]}_{q}}\right){\left(B_{3}\left(q;\frac{1}{{\left[3\right]}_{q}}\right){\left|{\;}^{\;\pi_{2}}D_{q}F\left(\;\pi_{1}\right)\right|}^{p}+C_{1}\left(q;\frac{1}{{\left[3\right]}_{q}}\right){\left|{\;}^{\;\pi_{2}}D_{q}F\left(\;\pi_{2}\right)\right|}^{p}\right)}^{\frac{1}{p}}\right.\\ & & \left.+A_{4}^{1-\frac{1}{p}}\left(q;\frac{1}{{\left[3\right]}_{q}}\right){\left(B_{4}\left(q;\frac{1}{{\left[3\right]}_{q}}\right){\left|{\;}^{\;\pi_{2}}D_{q}F\left(\;\pi_{1}\right)\right|}^{p}+C_{2}\left(q;\frac{1}{{\left[3\right]}_{q}}\right){\left|{\;}^{\;\pi_{2}}D_{q}F\left(\;\pi_{2}\right)\right|}^{p}\right)}^{\frac{1}{p}}\right\}. \end{array}$$

**Proposition** **6.** *Under the assumptions of Theorem 8 with $\;\varsigma =\frac{1}{{\left[4\right]}_{q}}$, we have*

$$\begin{array}{ccc} & & \left|\frac{{\left[2\right]}_{q}}{{\left[4\right]}_{q}}\left[\frac{F\left(\;\pi_{1}\right)+qF\left(\;\pi_{2}\right)}{{\left[2\right]}_{q}}+q^{2}F\left(\frac{\;\pi_{1}+q\;\pi_{2}}{{\left[2\right]}_{q}}\right)\right]-\frac{1}{\;\pi_{2}-\;\pi_{1}}\int_{\;\pi_{1}}^{\;\pi_{2}}F\left(\;\nu \right){\;}^{\;\pi_{2}}d_{q}\;\nu \right|\\ & \leq & \left(\;\pi_{2}-\;\pi_{1}\right)\left\{A_{3}^{1-\frac{1}{p}}\left(q;\frac{1}{{\left[4\right]}_{q}}\right){\left(B_{3}\left(q;\frac{1}{{\left[4\right]}_{q}}\right){\left|{\;}^{\;\pi_{2}}D_{q}F\left(\;\pi_{1}\right)\right|}^{p}+C_{1}\left(q;\frac{1}{{\left[34\right]}_{q}}\right){\left|{\;}^{\;\pi_{2}}D_{q}F\left(\;\pi_{2}\right)\right|}^{p}\right)}^{\frac{1}{p}}\right.\\ & & \left.+A_{4}^{1-\frac{1}{p}}\left(q;\frac{1}{{\left[4\right]}_{q}}\right){\left(B_{4}\left(q;\frac{1}{{\left[4\right]}_{q}}\right){\left|{\;}^{\;\pi_{2}}D_{q}F\left(\;\pi_{1}\right)\right|}^{p}+C_{2}\left(q;\frac{1}{{\left[4\right]}_{q}}\right){\left|{\;}^{\;\pi_{2}}D_{q}F\left(\;\pi_{2}\right)\right|}^{p}\right)}^{\frac{1}{p}}\right\}. \end{array}$$

## 5. Conclusions

In this investigation, we have proven a parameterized *q*-integral identity involving *q*-derivatives and then used this result to prove some new *q*-integral inequalities for differentiable convex functions. We also showed that the results established in this paper are a potential generalization of the existing comparable results in the literature. The results proved in this research can be used in quantum information theory, an interdisciplinary field that includes computer science, information theory, philosophy, cryptography, and entropy. As a future direction, similar inequalities could be found for co-ordinated convex functions.

## Data Availability

Not applicable.
